# Process Evaluation of an Implementation Trial: Design, Rationale, and Early Lessons Learnt From an International Cluster Clinical Trial in Intracerebral Hemorrhage

**DOI:** 10.3389/fmed.2022.813749

**Published:** 2022-06-15

**Authors:** Menglu Ouyang, Craig S. Anderson, Lili Song, Alejandra Malavera, Stephen Jan, Guojuan Cheng, Honglin Chu, Xin Hu, Lu Ma, Xiaoying Chen, Chao You, Hueiming Liu

**Affiliations:** ^1^The George Institute for Global Health, Faculty of Medicine, University of New South Wales, Sydney, NSW, Australia; ^2^The George Institute China at Peking University Health Science Center, Beijing, China; ^3^Neurology Department, Royal Prince Alfred Hospital, Sydney Health Partners, Sydney, NSW, Australia; ^4^Heart Health Research Center, Beijing, China; ^5^Research Center of Clinical Epidemiology, Peking University Third Hospital, Beijing, China; ^6^Department of Neurosurgery, West China Hospital, Sichuan University, Chengdu, China

**Keywords:** process evaluation, stroke, intracerebral hemorrhage, clinical trial, implementation science

## Abstract

**Background:**

The third INTEnsive care bundle with blood pressure Reduction in Acute Cerebral Hemorrhage Trial (INTERACT3) is an ongoing, international, multicenter, stepped-wedge cluster, prospective, randomized, open, blinded endpoint assessed trial evaluating the effectiveness of a quality improvement “care bundle” for the management of patients with acute spontaneous intracerebral hemorrhage (ICH) in low- and middle-income countries (LMICs). An embedded process evaluation aims to explore the uptake and implementation of the intervention, and understand the context and stakeholder perspectives, for interpreting the trial outcomes.

**Methodology:**

The design was informed by Normalization Process Theory and the UK Medical Research Council process evaluation guidance. Mixed methods are used to evaluate the implementation outcomes of fidelity, reach, dose, acceptability, appropriateness, adoption, sustainability, and relevant contextual factors and mechanisms affecting delivery of the care bundle. Semi-structured interviews and non-participant observations are conducted with the primary implementers (physicians and nurses) and patients/carers to explore how the care bundle was integrated into routine care. Focus group discussions are conducted with investigators and project operational staff to understand challenges and possible solutions in the organization of the trial. Data from observational records, surveys, routine monitoring data, field notes and case report forms, inform contextual factors, and adoption of the intervention. Purposive sampling of sites according to pre-specified criteria is used to achieve sample representativeness.

**Discussion:**

Implementation outcomes, and relevant barriers and facilitators to integrating the care bundle into routine practice, will be reported after completion of the process evaluation. The embedded process evaluation will aid understanding of the causal mechanisms between care bundle elements and clinical outcomes within complex health systems across diverse LMIC settings.

**Trial Registration:**

The INTERACT3 study is registered at ClinicalTrials.gov (NCT03209258).

## Introduction

Intracerebral hemorrhage (ICH) is the most severe and least treatable type of stroke, contributing to the significant global burden of disease (1) particularly in low- and middle-income countries (LMICs) (2, 3). Protocols to systematically monitor and control key physiological parameters such as blood pressure (BP) and blood glucose level, may improve the outcome in patients with acute ICH (4). The third INTEnsive care bundle with blood pressure Reduction in Acute Cerebral Hemorrhage Trial (INTERACT3) is an international, multicenter, stepped-wedge cluster, prospective, randomized, open, and blinded endpoint assessed trial which aims to determine the effectiveness of a quality improvement “care bundle” in patients with acute ICH in LMICs. This care bundle comprises early intensive BP lowering (achieving systolic BP <140 mmHg), glycemic control (achieving 6.1–7.8 mmol/L and 7.8–10.0 mmol/L without and with diabetes mellitus), treatment of pyrexia (achieving temperature level <37.5°C), and reversal of anticoagulation [achieving international normalized ratio (INR) <1.5], within 1 h of initiation of treatment and maintained for 7 days. The stepped-wedge study design requires a smaller sample size compared to a parallel-arm design but creates potential problems for retention due to time lags between recruitment and when the intervention starts, as well as potential confounding caused by variation in time. Therefore, it is important to ensure that the delivery of the care bundle is consistent across sites as planned (5). As a complex intervention with multiple components and involving organizational change, there is a need to provide details of how the care bundle is delivered and what local contextual factors impact outcomes (6, 7). Insufficient details of how the complex intervention, such as the care bundle in INTERACT3, and its components were implemented may limit the transferability of the evidence to other contexts, which is a recognized barrier to providing optimal care and treatment (8, 9). Moreover, consideration of how implementation can address knowledge gaps in real world settings can better inform potential sustainability and scale-up (10). The process evaluation of the INTERACT3 trial allows an examination of the complexities of implementation strategies, provides explanations for discrepancies between expected and observed outcomes, offers insights into how context influences outcomes, and aids in considering the potential for wider implementation (11–13).

A process evaluation (PE) was embedded into INTERACT3 with three principle aims: (i) determine implementation outcomes of the care bundle through fidelity (whether the care bundle was delivered as intended), dose (what quantity and quality was delivered), reach (whether all eligible ICH patients received all components), acceptability (whether the care bundle was agreeable and acceptable to participants), appropriateness (participant views on the perceived fit or relevance of the care bundle in their practice settings), and adoption and sustainability (whether the care bundle was integrated and incorporated within routine practice and local policies); (ii) provide information to explain the trial results regarding possible barriers and facilitators related to the implementation on each component of the care bundle, their integration into routine practice, and possible context factors; and (iii) determine transferability and sustainability of the care bundle in LMICs through provision of participant perspectives on how and why the care bundle can (or cannot) be implemented at a national level.

## Methods

### Study Design

**Figure 1:**
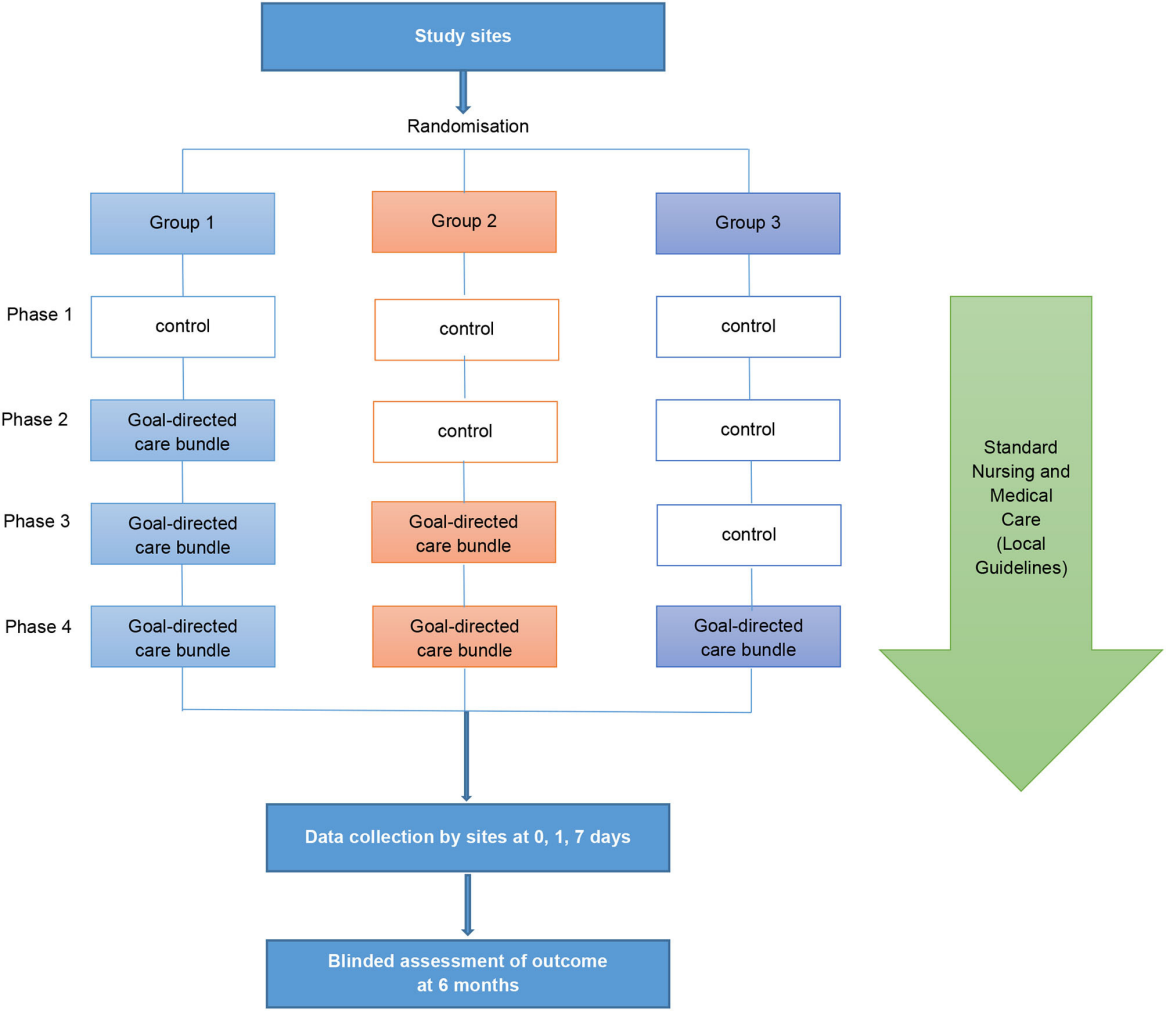
Stepped-wedge design of INTERACT3.

INTERACT3 is a cluster stepped-wedge design that aims to evaluate the effectiveness of a care bundle in 8,360 patients at 110 hospitals in 10 LMICs [Brazil, Chile (identified as a high-income country in 2021), China, India, Mexico, Nigeria, Pakistan, Peru, Sri Lanka, and Vietnam] from December 2017 to October 2022. The unit of randomization is the hospital sites, randomly assigned by a blinded statistician into three groups which undergo four phases (Figure 1). All sites start in a control “usual care” phase before being randomly allocated to transfer to the intervention phase where the care bundle protocol is to be implemented as part of the routine standard of care. The procedures of the trial intervention implementation are described in greater detail in Supplementary Appendix 1. The stepped-wedge cluster design allows implementation of the care bundle through a one direction cluster switch (from control to treatment) at different time points, reduces contamination between control and intervention patients, and allows evaluation of implementing multi-faceted system-wide changes (14). Details of the study design are described elsewhere (15).

### Theoretical Approach

**Figure 2:**
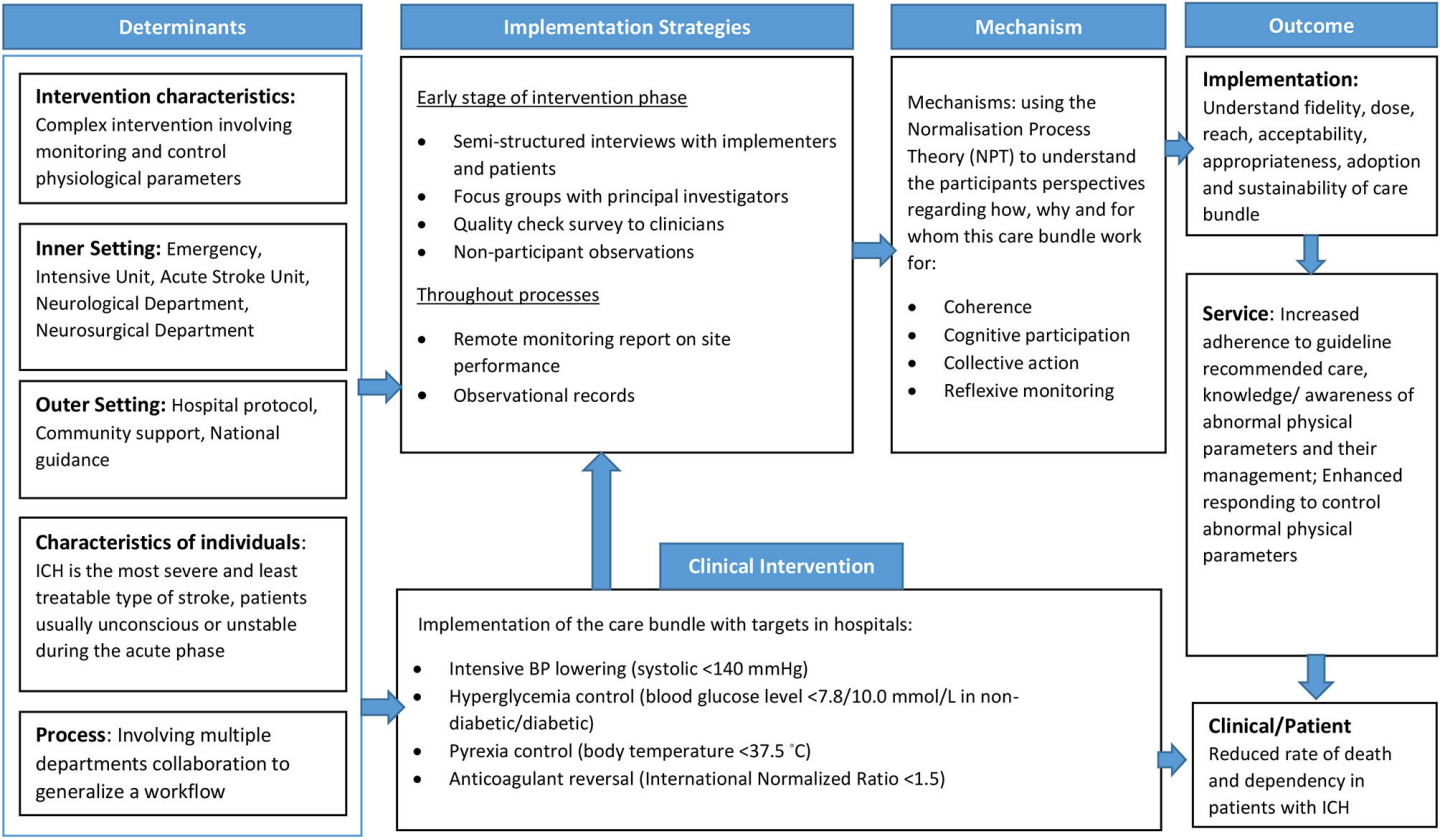
Implementation research logic model of INTERACT3 process evaluation.

The PE for INTERACT3 is informed by the UK Medical Research Council (MRC) (10) process evaluation guidance and Normalization Process Theory (NPT). The MRC guidance framework includes three main components of inquiry: implementation of the intervention, mechanisms of impact, and context to result in the trial outcomes. NPT is used to understand adoption and integration of the care bundle into routine medical care practice (16). It is an implementation science theory that provides a deeper understanding of embedding integration and sustainability of a new model of care or guidelines, and to enhance understanding of the outcome data (17). The core components of NPT include coherence, cognitive participation, collective action, and reflexive monitoring (18). A logic model of contextual determinants and intervention components was developed to describe how the care bundle and research activities result in short- and long-term outcomes, and to inform data collection of relevant process indicators (Figure 2). Considering the different contexts of each country, a separate implementation research logic model for each country is being generated to guide the PE (19).

### Setting

The study is being undertaken across different areas of hospitals, including emergency, acute stroke unit, intensive care unit, and neurology and neurosurgery areas/wards, where representative health professionals are being recruited through purposive sampling for a semi-structured interview, (20) stratified by country. With at least two sites being sampled for interviews in each participating country, it is estimated that 28–32 sites will be included covering geographical location (across regions), level of hospital (tertiary vs. secondary), department (neurosurgical vs. neurology or emergency), and performance (e.g., recruitment speed, and cooperation), although the final number will be determined by saturation of themes and available resources.

### Participants

Participants for the PE include key stakeholders involved in implementing the INTERACT3 intervention, such as study investigators, ward clinicians and nurses, patients (or carers) who have received the care bundle, and clinical research associates (CRAs) involved in training site staff in delivering the intervention. An information sheet and consent form will be sent to potential participants about the PE and inviting them to an interview and/or focus group discussion with a member of the PE team.

### Data Collection

A parallel mixed-method approach is designed for data collection, (21) to provide different perspectives, validation, and triangulation from multiple sources (11). Tables 1, 2 outline the approach for both qualitative (semi-structured interviews, focused groups discussion, non-participant observation) and quantitative (surveys) methods to explore implementation outcomes. Other data sources include observational records and a hospital organization questionnaire to provide additional context for the participating sites and inform the sampling frame. The time point for data collection will be at an early phase of intervention, ideally after 2–5 patients have been enrolled, in order to obtain feedback from site staff about implementation challenges and allow the operations team to better support sites to optimize implementation of the care bundle.

**Table 1:** Summary of data collection methods.

**Item**	**Data collection method**	**Participants**	**Participant number**	**Time point of data collection**
1	Semi-structured interviews	Clinicians and nurses from selected sites	3–4 sampled sites	At early stage of intervention phase (e.g., 5–10 enrolled intervention patients)
2	Semi-structured interviews	Patients/carers from selected sites	2–3 sampled sites	Patients with a stable condition (before discharge) in the intervention group
3	Non-participant observations	Selected sites	Purposive sampling sites (assume 16–20)	Onsite monitoring visit
4	Focus group discussions	PI invited	Purposive sampling sites (assume 16–20)	Investigator meetings and quality control meetings
5	Focus group discussions	All CRAs	Purposive sampling sites (assume 16–20)	At the early phase of the intervention phase
6	Questionnaires	Clinicians		
7	Survey	Clinicians and nurses	All sites	Quality control meetings and at the time of study close out
8	Monitoring records, including routine monitoring data, field notes, recruitment logs, and case report forms	N/A	All sites	Throughout the study

**Table 2:** Implementation outcomes summary.

**Implementation outcomes**	**Aims**	**Data sources**
Fidelity	Whether the care bundle under investigation in INTERACT3 was delivered as intended	Semi-structured interviews Non-participant observations Surveys Routine monitoring data, field notes, and case report forms
Dose	What the quantity and quality of the care bundle and each component delivered were	Semi-structured interviews Surveys Routine monitoring data, field notes, and case report forms
Reach	Whether all eligible patients received all components of the care bundle	Semi-structured interviews Non-participant observations Recruitment logs
Acceptability	Whether the care bundle was agreeable and acceptable to participants	Semi-structured interviews Focus group discussions Survey
Appropriateness	Participant views on the perceived fit or relevance of the care bundle in their practice settings	Semi-structured interviews Focus group discussions
Adoption and sustainability	Whether the care bundle was integrated and incorporated into routine practice and local policies	Semi-structured interviews Non-participant observations Focus group discussions Routine monitoring data, field notes, and case report forms

#### Semi-structured Interviews

Semi-structured interviews will be conducted with implementers (physicians and nurses) and patients/carers from purposively sampled sites at an early phase of the intervention. At each sampled site, 3–4 implementers and 1–2 patients/carers are invited to interview. For the implementers, the evaluation will explore options on challenges to implement the intervention, facilitating factors, context, progress on implementation, and perspectives of the intervention. For patients/carers, the interview will focus on their perspectives of receiving the goal-directed care bundle, and their thoughts and concerns about participating in the study. Only patients who are medically stable will be invited to participate in an interview. The timing of the patient interview will be at hospital discharge (face to face) or during their follow up (*via* telephone) according to the patient's conditions and request. A semi-structured interview guide (see Appendix 2 in Supplementary Material) has been developed, based on the objectives of the PE and after pilot testing. Early findings from interviews are discussed with the project operation team to allow any modifications to procedures. Trained interviewers collect the qualitative data under the supervision of an experienced qualitative researcher by a face-to-face or teleconference interview.

#### Focus Group Discussion

Focus group discussions (see Appendix 3 in Supplementary Material) are conducted to explore contextual factors and implementation barriers of the care bundle as part of an international collaboration. Two sets of focus group discussions are conducted, involving the clinical trial coordinating team and principal investigators (PIs) or sub-principal investigators (Sub-Is) from selected sites. For the former, the group discussion will mainly involve CRAs to evaluate how well the training sessions were delivered to site implementers and received and how well they find and assist in overcoming the barriers in covering presentations, on-site monitoring visits, and communications and interactions with implementers. The focus group discussions involving national PIs and Sub-Is from participating sites aim to identify barriers at coordinating the site, including roles and responsibilities, leadership, staff training, and in providing daily support. These discussions are facilitated by the PE team from the International Coordinating Center *via* teleconference.

#### Non-participant Observation

The non-participant observation (see Appendix 4 in Supplementary Material) aims to understand contextual factors, recruitment processes, and delivery of the care bundle. An observation template was adapted from a PE for another stroke trial (22) to allow collection of information on implementer behavior of operational staff alongside an on-site monitoring visit. Trained observers from the Regional Coordinating Center conduct the observation at the purposively sampled site.

#### Survey

A quality check survey informed by NPT is used to collect perceptions of the intervention and other relevant information from clinicians (see Appendix 4 in Supplementary Material) during the intervention phase. The quality check survey has been piloted at meetings with investigators from 20 sites who had completed the initial vanguard phase in China. All sites outside of China are invited to complete the survey at an early phase of intervention as a part of the PE.

Contextual information of health services are collected through a Hospital Organization Questionnaire (HOQ) sent to all sites prior to patient recruitment (see Appendix 6 in Supplementary Material). Monitoring records, field notes, and case report forms are obtained to allow an evaluation of whether the intervention has been delivered as intended. These quantitative data are reviewed monthly as part of routine monitoring of patient recruitment, data quality, and adherence to the protocol. Monthly performance reports, highlighting recruitment targets and details of protocol adherence and intervention implementation, will also be retrieved to assist sampling of the participating sites.

### Data Management

Data will be stored electronically in a secure password-protected system only accessible to specified members of the research team. Interview transcripts will be uploaded into the software program NVivo V.9 for data analysis.

### Analysis and Report

Qualitative data from focus groups, semi-structure interviews, non-participant observations, and free text answers to sections of the survey will be thematically analyzed (23, 24). Inductive findings of the interviews involving the first three sites will be discussed by the PE team to explore emerging themes to guide subsequent interviews. Interim analysis will be performed after 5–10 interviews to further adapt the interview format and to generate themes for subsequent interviews. The data will be independently coded by two trained researchers using a coding framework developed through iterative input from investigators to reveal consistency in patterns of data. Descriptive statistics will be undertaken on the survey data, with frequencies and percentages used to summarize categorical variables, and for means or median reported for continuous variables. Analysis will be initially stratified by the country to understand local context, and to co-design implementation strategies for that context. The integration of quantitative and qualitative data will be done through merging and comparison across the numerical and textual data, addressing similar research questions (25). Reporting of the integrated data will be done through a mixed methods joint display (26) that synthesizes data with a visual display and summarizes the meta-inference of the findings.

The qualitative findings will be reported in accordance with the consolidated criteria for reporting qualitative research (COREQ), (27) with the implementation outcomes used to monitor and document fidelity to the project plan.

## Discussion

LMICs face different barriers in implementing interventions in comparison to high-income countries (HICs), such as limited human resources, limited access to health care, and limited skills of healthcare providers (28). The emerging issues of acute care for stroke in LMICs are often relate to the limited health systems. For example, compared to HICs, stroke care units are less common in LMICs and relevant acute care treatment such as intensive blood pressure reduction are seldom offered (29). In addition, in other LMIC settings, such as in Africa, stroke patients are cared for by non-specialized health providers without the support of a multidisciplinary team due to a lack of allied health professionals such as physiotherapists and speech therapists (30). PE is crucial to understanding contextual factors that may impact intervention implementation, especially as to whether the intervention can be adapted and implemented effectively across other contexts in LMICs (13). Contextual factors (COVID-19 impact, current policies, and settings resources, etc.) that could influence delivery of the intervention can be identified through interviews, focus group discussions, observations, and survey to enable a better understanding of the results, and the opportunity for future scale-up of the intervention to other LMICs. The INTERACT3 PE aims to inform a broader implementation plan that can be tailored to local contextual factors to improve the quality of care for patients with ICH, the most severe type of stroke. Relevant data pertaining to local stroke protocols and care pathways will provide a useful assessment of health systems for planning further studies that incorporate PE to strengthen the implementation and assessment of complex health service interventions in multicenter clinical trials that include participation from LMICs.

A systematic review of the use of PE in translational research indicates that most evaluations involve data collection at the post-intervention phase, but which has limited value in optimizing implementation of the trial in complex health systems (31). In INTERACT3, we have taken the opportunity to conduct a PE at the early phase of the intervention to assist in the timely identification of barriers and facilitators, to allow the coordinating team to address any issues that arise, and to foster clinician confidence through support and training (17). For example, in some earlier interviews, we found a shortage of suitable antihypertensives, which then had to be budgeted for and advocated for by the project operation team. Moreover, implementation outcomes will also be useful in explaining what was actually done in real world settings and allow a better unpacking of any potential variation in the proposed treatment effect under investigation in the trial. The causal relationship between the intervention and trial outcome in real-life implementation might be affected by adaptability/unpredictable actors and by a wide range of influencing elements at geographical and organizational levels (e.g., the impact of the COVID-19 pandemic on workforce capacity, and patient engagement with health services).

### Strength and Weakness of Our Process Evaluation Design

Key strengths of this study include the use of multiple methods and diverse sources of data to obtain a comprehensive picture of the implementation of a goal-directed care bundle. Mixed methods evaluations draw upon strengths of both qualitative and quantitative approaches to provide a more holistic understanding of multi-level processes and the nature of an individual's experience (25). The PE has been conducted across different health care systems in multiple countries to document variable care pathways and health system factors (e.g., workforce, medication availability), and to assist in understanding the value of implementation research and its generalizability. However, there are limitations such as selection and information bias due to voluntary participation in interviews, which is further influenced by the COVID-19 pandemic in restricting on-site visits for patient interviews and observations, and the need to conduct many interviews by teleconference/video conference. Data provided through remote monitoring and regional coordinating data can go some way in mitigating these issues. In addition, the survey focuses on barriers in embedding the care bundle into routine care that can result in biased answers, without the opportunity for positive feedback, which we aim to amend in future trials. Even flexible time points were offered for patient interviews, this may have introduced recall bias in relation to patient-reported experiences of the care bundle.

### Timeline of the PE

The PE is being undertaken in stages and will be completed within 6 months of sites being activated in participating countries. However, due to the emergence of the COVID-19 pandemic in early 2020, timelines were extended in China since patient recruitment, transfer to intervention phase, and project staffing resources were all affected. The PE in other countries commenced in 2021, but again progress depends on the degree of the ongoing COVID-19 impact in each country.

### Trial and PE Status

In October 2021, there were 5,986 patients recruited into INTERACT3, including 261 enrolled outside of China. In China, focus group discussions involved 14 investigators from 9 sites, and 24 interviews with doctors/nurses at 9 sites, during January to December 2020. Preliminary findings of the PE in China have been reported to the project team to enhance daily operation and monitoring. In other participating countries, semi-structured interviews and focus group discussions have been completed in Chile and Peru. Due to the ongoing nationwide strike of health workers in Nigeria from August 2021, recruitment and PE have paused until the situation changes.

### Reflections

The PE in our large multicenter international clinical stroke trial has improved capacity building at regional coordinating centers in their qualitative research skills for conducting interviews and observations. However, the involvement of multiple countries requires significant ongoing efforts to address local language and cultural barriers. Although this has been time and resource intensive as a crucial component of the PE, it has strengthened international collaborations through sharing experiences. For example, contextual determinants such as medication supply shortage in rural centers in China and the delay in the ED to obtain a timely scan in Nigeria were discussed with the project operation teams in order to improve the implementation. However, in hindsight, some of these barriers could have been identified previously. Therefore, we recommend the collection of preliminary data prior to intervention delivery across countries to better understand local health systems and inform focus group discussions. This could be facilitated by co-developing an implementation logic model and implementation strategies to overcome anticipated local barriers with the local PIs and the trial coordinating team.

## Ethics and Dissemination

Ethical approval for this study has been obtained from central and site-specific ethic committees in each country. The information sheet will be provided to the participants prior to individual interviews and focus group discussions. Written consent will be obtained prior to interviews and verbal consent will also be taken prior to any participation in a focus group discussion.

## Conclusions

The PE of the INTERACT3 study will not only provide insights necessary to optimize implementation of the care bundle intervention across diverse settings in LMICs, but it will also lead to better understanding of the relationship between elements of the care bundle and outcomes. Our embedded PE will advance the future conduct of international pragmatic stroke clinical trials to optimize intervention implementation within complex health system contexts.

## Data Availability Statement

Datasets generated and/or analyzed for INTERACT3 will be available to all study investigators, and investigators from other institutions around the world, according to a strict data sharing agreement. Data sharing will be available from 12 months after publication of the main results. Investigators are to make a formal request for data sharing through the Research Office of The George Institute. Access will be controlled by the Principal Investigators, with the approval of the Trial Steering Committee.

## Ethics Statement

All written informed consent to participate in the study were obtained. The Biomedical Ethics Committee of West China Hospital approved the INTERACT3 study before the commencement of any patient recruitment (Ethics Reference No. 22017 Review [217]). According to funding request from Medical Research Council, additional approval (Ethic Reference: 26596-tgr2r-ls: cardiovascular sciences, deptof) had been obtained from Research Ethics Committee of the University of Leicester, United Kingdom. Ethics approval was obtained in each site before site activation. The patients/participants provided their written informed consent to participate in this study.

## Author Contributions

HL, SJ, LS, and MO contributed to the concept and rationale for the study. MO wrote the first draft of manuscript with input from HL and CA. All authors contributed to the article and approved the submitted version.

## Funding

The INTERACT3 study is funded by the West China Hospital Outstanding Discipline Development 1-3-5 Program (ZY2016102), Program Grant from the National Health and Medical Research Council (NHMRC) of Australia (APP1149987) and Joint Global Health Trials grant (MR/T005009/1) from National Institute of Health Research (NIHR), The Department for International Development (DFID), The Global Challenges Research Fund (GCRF), and the Medical Research Council (MRC).

## Conflict of Interest

CA and LS declare that they received speaker fees and travel reimbursement from Takeda. The remaining authors declare that the research was conducted in the absence of any commercial or financial relationships that could be construed as a potential conflict of interest.

## Publisher's Note

All claims expressed in this article are solely those of the authors and do not necessarily represent those of their affiliated organizations, or those of the publisher, the editors and the reviewers. Any product that may be evaluated in this article, or claim that may be made by its manufacturer, is not guaranteed or endorsed by the publisher.
