# Supplementary material for: Process Evaluation of an Implementation Trial: Design, Rationale, and Early Lessons Learnt From an International Cluster Clinical Trial in Intracerebral Hemorrhage
Source: Front Med (Lausanne). 2022 Jun 15;9:813749. doi: 10.3389/fmed.2022.813749 (PMC9240283; doi:10.3389/fmed.2022.813749)
Supplement: Supplementary file 1 [file Data_Sheet_1.PDF]

Supplemental Material

Process evaluation of an implementation trial: Design, rationale, and early lessons learnt from an international cluster clinical trial in intracerebral hemorrhage

Menglu Ouyang,[1] Craig S. Anderson,[1,2,3,4] Lili Song,[1,2] Alejandra Malavera,[1] Stephen Jan,[1] Guojuan Cheng,[2] Honglin Chu,[5] Xin Hu,[6] Lu Ma,[6] Xiaoying Chen,[1] Chao You,[6*] Hueiming Liu[1*]

[1]The George Institute for Global Health, Faculty of Medicine, University of New South Wales, Sydney, Australia

[2]The George Institute China at Peking University Health Science Centre, Beijing, China

[3]Neurology Department, Royal Prince Alfred Hospital, Sydney Health Partners, Sydney, Australia

[4]Heart Health Research Centre, Beijing, China

[5]Research Center of Clinical Epidemiology, Peking University Third Hospital, Beijing, China

[6]Department of Neurosurgery, West China Hospital, Sichuan University, NO.43, St. Guoxuexiang, Chengdu, China

***Corresponding Authors**

Appendix 1. Implementation of the trial procedures

The unit of randomization is the hospital site, randomly assigned by a statistician not otherwise involved in the study using a pre-specified randomization schedule with permuted blocks. Participating sites are stratified according to country and the estimated recruitment capacity (ranging from 40,80,120,160 and 200).

In phase 1, all hospitals will be observed under usual care “control” conditions according to usual management of ICH patients. In phase 2, the first cluster of hospitals (group 1) will start implementing the intervention (care bundle), and then sequentially, groups 2 and 3 will start implementing the interventional package in phases 3 and 4, respectively, so that by phase 4, all hospitals will be receiving the intervention, with those in group 1 having the intervention for longest and those in group 3, the shortest. Once the site has completed the necessary control usual care observational period, they will receive training on the application of the care bundle intervention during the study. The site investigator will be informed to pause enrolment when the patient number or study duration in control phase has reached the pre-specified target, and then will be instructed to transfer over to the intervention phase after a recruitment interval of 7–10 days.

Appendix 2. Semi-structured interview guide

Implementer interview guide

Section One
Warm-up questions:<br>• Name<br>• What is your role at the hospital, and what are your main responsibilities?
Section Two
Perceptions about the intervention (NPT domain: coherence)<br>• How did you hear about INTERACT3? Did the PI tell you about it?<br>• What did you think was the aim of the INTERACT3 study? (probes: any concerns regarding clinical aspects of the intensive blood pressure reduction in stroke care? any clinical concerns regarding the care bundle in INTERACT3?)<br>• When you compare the care bundle in INTERACT3 and routine care in your hospital, which one was easier to deliver? Please explain why.<br>• What do you think about the training provided by CRAs for this INTERACT 3 study? (probe: did we need to more training once you were in the intervention phase?)
Section Three
Implementation/delivery and actions needed (NPT domain:cognitive participation and collective action)<br><u>Reach</u><br>• How have you found patient recruitment? Have you had any difficulties in recruiting patients for INTERACT3 at your hospital? Please explain why and can you give me an example?<br>• Did you recruit all eligible patients? Why or why not?<br><br><u>Intervention Fidelity</u><br>• How was the cross over to the INTERACT care bundle? Did you experience any difficulties? (probes: staff understanding or concerns, patients' and carers' understanding or concerns, or other?)

- Did the patients receive all the components of care bundle? (probe: If not, please explain why, separate components of BP lowering, glycaminc control, treatment of pyrexia, and reveral of anticoagulation).

- You have since recruited 5-10 patients, has there been any changes in how you implemented the care bundle? (probe: separate components)

- How well were the care bundle targets reached in the first hour when abnormal blood pressure, blood sugar level, body temperature and INR were detected? (Probe: reasons for not achieving the targets, provide an example)

<u>Implementation</u>

- What difficulties have you experienced in implementing INTERACT3 so far?
  (i.e. Such as paperwork, data entry, communication with other health professionals/departments, equipment, What were the patients' and their carers responses when you implemented the care bundle?)
- What could we do to support the implementation of INTERACT 3 at your hospital? (probe: BP lowering guidance, data entry)

<u>Context/external factors</u>

- Were there any external factors that affected the implementation of the care bundle to the patients? (e.g. availability of medication, any changes or events at your hospital during INTERACT3 that impacted on participation or recruitment?
- If we want to implement the care bundle as a guideline widely in hospitals across your country, what would be the main barriers ? Please explain.

Section Four
Perceived effects (NPT domain: reflexive monitoring)<br>• How well do you think INTERACT3 worked overall in your hospital?

• What is the role of PI? What was done by PI regarding the project process? (probe: What additional support could have helped from PI or from the clinical trial team?)<br>• Do you have any suggestions on how to improve recruitment and implementation of the INTERACT 3 bundle? (probe: do they anticipate any further issues)<br>• Concluding question: Do you have any other thoughts about INTERACT3 besides what we talked about above?<br>(Thank you so much for your participation in this research and for sharing your insights)

Patient interview guide

Purpose of the interview: ***understand patient/carer perspectives of the goal-directed care bundle, especially what intervention was actually received, thoughts and cooperation of increased monitoring and concerns of participating in INTERACT3 trial.***

Questions classification:

- *Initial broad descriptive questions*
- o *Probing questions are guide only, depends on patients response on the broad questions. Don't have to ask all these questions exactly as written*

Section One
Health care experience<br>• Can you tell me about your stroke?<br>• How was the care in the hospital you received?<br>• How have you been since being discharged? Have you assessed any rehabilitation services and follow up with health providers?<br><br>Probing questions:<br>o What are some of the good/bad things about your health care?<br>o What type of support you get from family, community or social groups with looking after your health?<br>o What kind of roles/responsibilities do you have in your family?
Section Two
Perspectives on the goal-directed care bundle<br>• What are your thoughts about your current treatment for your stroke?<br>• Can you describe the treatment you have received?<br><br>Probing questions:<br>o What is the frequency of your blood pressure/blood glucose level/body temperature monitoring? Who is providing these monitor? Do you think it is okay to have this kind of monitoring?<br>o What was good of the treatment?<br>o What was not helpful?<br>o How can your doctor or the treatment team improve your current care?<br>o What has been most important in helping you getting better?

Section Three
<u>Specific components of the care bundle if not covered</u><br>• Can you tell me your understanding of following care in specific:<br>-intensive blood pressure lowering<br>-glycaemic control<br>-fever control<br>-reversal of anticoagulation<br><br>Probing questions:<br>o Based on your understanding, do you have any concerns of intensive blood pressure reduction?<br>o What is your thought of regular monitoring glucose level?<br>o Can you describe any time or situation when you didn't want to receive the care bundle?<br><br><u>Cooperation</u><br>• What is your cooperation with health care providers of receiving the care bundle?<br><br>Probing questions:<br>o Can you describe any time or situation when you didn't want to cooperate with doctors/nurses for your treatment?
Section Four
<u>General views about the trial</u><br>• What are your thoughts about INTERACT3 trial in general?<br><br>Probing questions:<br>o Tell me how you think the study worked and what it was hoping to achieve?
<u>Recruitment/participating</u><br>• Tell me about your thoughts of participating in this trial<br>• What is being informed for participating in this trial?<br><br>Probing questions:<br>o What were the things that made you want to participate in the study initially?<br>o What were the benefits/concerns to you of participating in this study?<br>o Did you know there will be increased monitoring in the bundle care?<br>o Were there any things that may have stopped you from participating initially?<br>o Did you feel that you could withdraw at any time?<br>o Did you know whom to contact if you had any concerns about the trial?<br>o What were your thoughts about your privacy throughout this study?
Section Five
<u>Concluding questions</u><br>• Are there things which we can do better to improve the study or the running trial?<br>• Is there something else your would like to say if we have not covered in this interview?
**Thanks for your time!**

Appendix 3. Focus group discussion guide

Introduction: This focus group discussion is designed to assess your current thoughts and feelings about the intervention implementation in INTERACT3 project. The focus group discussion will take no more than 1.5 hour.

Anonymity: The discussion will be audio-recorded. I would like to assure you that the discussion will be anonymous. The record will be kept safely in a locked facility until they are transcribed word for word, then they will be destroyed. The transcribed notes of the focus group will contain no information that would allow individual subjects to be linked to specific statements. You should try to answer and comment as accurately and truthfully as possible. I and the other focus group participants would appreciate it if you would refrain from discussing the comments of other group members outside the focus group. If there are any questions or discussions that you do not wish to answer or participate in, you do not have to do so; however please try to answer and be as involved as possible.

Information consent: Before we start our discussion, I would like you to express your decision to participate. If you are fully understand above information and agree to participate in this interview, please repeat:

"I fully understand the background, aim, procedure, risk and benefit of participating the discussion for INTERACT3 process evaluation. I have enough time and chance to raise questions and satisfied with the answers. I agree to participate in this discussion. I acknowledge I can reject to participate at any time with no reason."

Introductory question

I am just going to give you a couple of minutes to think about your experience of participating the INTERACT3 project. Is anyone happy to share his or her experience?

Guiding questions for CRAs

- What are the attitudes of you and clinicians towards the intervention from your training and communication? (What did clinician think/say/do?)
- What drove the positive/negative reaction? If negative, how could it be rectified?
- What has been challenging

  (e.g. any difficulties did you have to deliver trainings for physicians? any difficulties did you meet when monitoring the sites (include daily monitor, remote monitor and on-site monitor)?
- How many hours did you spend for INTERACT3 training (include on-site training, remote training via phones, email or messages)? How well did you think of the training you provided to the implementers (those who recruit patients and implement care bundle)?

- What are the physician's aspects / feedbacks on how and why to implement the care bundle and for whom the care bundle works for? (any concerns regarding to the interventions implementation?)
- Does the physicians involve in the program over time? If they withdrawal, what was the reason?
- Were there any barriers or facilitators did you think affect the implementation of care bundle by physicians? *Make examples and explain. (have you noted any difference across sites?)*
- *How can we support you in your role better?*

Guiding questions for PIs

- What are your attitudes towards the interventions in INTERACT3? (What did clinician think/say/do?)
- What do you think about the aims of having the care bundle for ICH patients?
- Do you think the intervention will improve the outcome of patients? If not, why not? (specified in each components of the intervention)
- What are the main issues around implementation of the care bundle in your ward? (implementation process, cooperation with ED, interaction between clinicians and nurses) What are the barriers to implement? What are the enablers?
- How far will existing work practices and the division of labor have to be changed or adapted to implement the intervention?
- Is any challenges you met when conducting the project in your ward (explore patient involvement, clinician teamwork and communication, data collection)? Are there any factors we can assist with?
- Did you feel comfortable to implement this intervention as routine care? Do you think this intervention can be widely used at a national level? Is the intervention consistent with the workplace and overall organization?

Concluding question

- Of all the things we've discussed today, what would you say are the most important issues you would like to express about the implementation? Are there any other things you would like to raise about the INTERACT3 that we haven't covered?

Appendix 4. Non-participant Observation Template

Name of observer:

Date:

Length of time of observation:

Site No. / Location(s) of observation:

Trial implementation	Description	Comments
Enrolment procedure		
-if eligible patients being recruited		
-inform consent procedure (any difficulties in these procedures? e.g. patients concerns, family concerns etc.)		
Care bundle (intervention)		
-workload (number of patients, number of ward rounds, number of observations or inspections on patients)		
-interactions/ communication with patients/family surrogates		
-interactions between the clinicians and nurses<br>-Is care bundle implementation in accordance with the order prescribed by clinicians?		
-what intervention were delivered?		

-Observe and record the procedure of blood pressure lowering/blood glucose control/temperature control/anticoagulant reversal<br>-Is the intervention implemented in accordance with the protocol?<br>-any barriers to implement each components of the complex interventions (etc. medication use, equipment, staffs)		
-what reactions of the patients regarding to the interventions implementation<br>-patient cooperation		

Appendix 5. Quality Control Survey

**Position (select one only).**
Resident
Attending physician ☐
Deputy chief physician ☐
Chief physician ☐
Nurse ☐
**2. Department**
Neurosurgery ☐
Neurology ☐
Neurological Intensive Care Unit(NICU) ☐
Emergency Department ☐
Others ☐
Please specify___________________________
**3. Working years**
< 1year ☐
1-6 years ☐
6-10 years ☐
More than 10 years ☐
**4. Do you have any experience in research (before INTERACT3)?**
Yes ☐
No ☐
4.1 If yes, how many clinical trial involved?
|__|__|

**5. Your role in INTERACT3 (*you can choose more than one option*).**	
PI	☐
Sub-PI	☐
Study coordinator	☐
Attending physician	☐
Research nurse	☐
Others	☐
Please specify___________________________	
**5.1 Please specify your familiarity of the trial protocol:**	
Very familiar	☐
Familiar	☐
Uncertain	☐
Unfamiliar	☐
Very unfamiliar	☐
**6. How would you rate the conduct of INTERACT3 at your site?**	
Very good	☐
Good	☐
Uncertain	☐
Not good	☐
Very bad	☐
6.1 *Please explain why:* ____	

**7. Has your treatment and management of ICH changed after participating in INTERACT3?**
Yes ☐   No ☐
7.1 *If yes, please specify which part has changed （you can choose more than one option）*
Blood pressure control ☐
Glycemic control ☐
Body temperature control ☐
Anticoagulation reversal ☐
Others ☐
Please specify：
**8. Are all eligible patients enrolled in INTERACT3?**
Yes ☐   No ☐
8.1 If no，which kind of patients were not enrolled*?*
Patient underwent surgery ☐
Severe patient ☐
Patient unable to do inform consent☐
Others ☐
Please specify
**9.Is there any delay of providing the intervention？**

Yes ☐	No ☐
9.1 If yes, what is the reason for delay (you can choose more than one option)?	
Patient underwent surgery	☐
Long stay at Emergency	☐
Others	☐
Please specify	
The following questions help us understand your concepts on intervention.	
**10. Regarding the target of intensive blood pressure lowering (reduce to 140mmHg within 1 hour and maintain for 7 days/before discharge), do you think it is easy to achieve?**	
Very Easy	☐
Easy	☐
Unsure	☐
Hard	☐
Very hard	☐
**11. What is the barrier to intensive blood pressure lowering** *(you can choose more than one option)*	
No ideal procedure	☐
Patients blood pressure hard to control	☐
Medication limitation	☐

Concerns of adverse effect	☐
Physicians unfamiliar with protocol	☐
Others	☐
Please specify	
**12. What do you think of the procedure of intensive blood pressure lowering in your department?**	
Very satisfied	☐
Satisfied	☐
Unsure	☐
Not satisfied, can be improved	☐
Very unsatisfied and hard to improve	☐
Please specify what need to be improved in detail	
**13. Regarding the target of glycemic control (non-diabetic patient maintain blood glucose level [BGL] at 6.1 to 7.8mmol/L while diabetic patients at 7.8-10.0mmol/L), do you think it is easy to achieve?**	
Very Easy	☐
Easy	☐
Unsure	☐
Hard	☐
Very hard	☐
**14. What is the barrier to achieve the target of glycemic control? (tick more than one)**	

Considering stress hyperglycemia therefore did not control ☐
Patients BGL hard to control ☐
Physicians unfamiliar with protocol ☐
Others ☐
Please specify
*14.1．For patients with increased glycaemia（**non-diabetic patients>=7.8mmol/L, diabetic patients>=10.0mmol/L**）,what is the reason for not commencing glycemic control immediately：*
Considering stress hyperglycemia ☐
Patient not cooperated ☐
Not control glycaemia routinely ☐
Others ☐
Please specify__________________________
**15. What do you think the procedure of glycemic control in your department?**
Very satisfied ☐
Satisfied ☐
Unsure ☐
Not satisfied, can be improved ☐
Very unsatisfied and hard to improve ☐
Please specify what need to be improved in detail
**16. Regarding the target of body temperature control (<37.5 C), do you think it is easy to achieve**
Very Easy ☐
Easy ☐
Unsure ☐
Hard ☐
Very hard ☐

**17. Regarding the intervention of anticoagulation reversal, do you think it is easy to implement ?**
Very Easy ☐
Easy ☐
Unsure ☐
Hard ☐
Very hard ☐
**18. What are the factors that impede the implementation of the goal-directed care bundle?**
**19. Is there any unexpected serious adverse event when implementing the care bundle?**
Yes ☐ No ☐
19.1If yes， did you report the event？
Yes ☐ No ☐
Please specify the event：
**20. Did you have any difficulty when change over from control to intervention phase?**
Yes ☐ No ☐
If yes, please specify
**21. Did you experience difficulty in the following aspects when you conducting INTERACT3? (you can tick more than one)**

Data collection	Yes ☐
Data entering	Yes ☐
Communicate with other staffs in the department	Yes ☐
Research equipment	Yes ☐
Concerns of the intervention	Yes ☐
Information consent	Yes ☐
Others	Yes ☐
Please specify :	
**22. If you have experienced problems in the implementation of the trial, what could we do to support you better? (you can tick more than one)**	
Additional site training	Yes ☐
Availability of research staff to call- when experiencing issues	Yes ☐
Online resources	Yes ☐
More frequent site visits	Yes ☐
Others	Yes ☐
Please specify:	

Appendix 6. Hospital Organisation Questionnaire

Name :	Hospital :
Address :	
Telephone :	
E-mail :	Department :

**1. General information**		
1.1	☐Y ☐N	Is the hospital a teaching hospital?
1.2	Location of the hospital: *(tick one answer)*	
	☐Y	Metropolitan / urban
	☐Y	Semi-metropolitan / semi-urban
	☐Y	Rural / countryside
1.3	Level of hospital: *(tick one answer)*	
	☐Y	Primary hospital
	☐Y	Secondary hospital
	☐Y	Tertiary hospital
1.4	☐Y ☐N	Do you have research experience in clinical trials?
		If had, please specify:

1.5	☐Y ☐N	Is your department participating in research of ICH? Especially research involves one or more following interventions: blood pressure (BP) reduction, blood glucose (BG) level control, body temperature control, dysfunction of coagulation regulation.
1.6	☐Y ☐N	Do you have experience of research with online data collection?
1.7.1	☐Y ☐N	Are you familiar with National Institute of Health Stroke Scale (NIHSS) assessment?
1.7.2	☐Y ☐N	Are your staff familiar with National Institute of Health Stroke Scale (NIHSS) assessment?
1.8.1	☐Y ☐N	Are you familiar with modified Rankin Scale (mRS) assessment?
1.8.2	☐Y ☐N	Are your staff familiar with modified Rankin Scale (mRS) assessment?
1.9.1	☐Y ☐N	Did you received NIHSS assessment training before?
1.9.2	☐Y ☐N	Did your staff received NIHSS assessment training before?
1.10	☐Y ☐N	Do you have certificate of NIHSS assessment?
1.11	☐Y ☐N	Does your hospital have a dedicated acute stroke unit?
1.11.1	|__|__| beds	If yes, number of beds in the stroke unit?
**2. Intracerebral Haemorrhage (ICH) related information**		
2.1	|__|__|__|__|	How many spontaneous ICH patients admitted in your department last year (except intracerebral aneurysm and AVM)?

2.2	|__|__| %	Proportion of admitted ICH patients with time of ICH stroke onset to admission **≤ 6 hours**
2.3	|__|__|__|	How many ICH patients received surgery treatment?
2.4	☐Y ☐N	Are the intracerebral haemorrhage (ICH) patients admitted into the hospital through Emergency Department (ED)?
2.4.1	|__|__|hours	If Yes, how long will the patients stay in the ED?
2.5	Which of stroke ward/unit that is in the hospital for ICH care:	
	☐Y	Acute stroke unit *(ie accept patients acutely but aims for early discharge (usually within seven days))*
	☐Y	Rehabilitation stroke unit *(ie accept patients after a delay, usually 7 or more days, with a focus on rehabilitation)*
	☐Y	Comprehensive stroke units *(ie combined acute and rehabilitation, accept patients acutely but also provides early rehabilitation for up to several weeks if necessary)*
2.5.1	What is the kind of care provided (are respondents to choose 1 option or all that applies?):	
	☐Y	'**Intensive**' with continuous monitoring, high staffing levels and life support facilities
	☐Y	**'Semi-intensive'** with continuous monitoring, high staffing but no life support facilities
	☐Y	**'Non-intensive'** with none of the above.
**3. Investigations for diagnosis and monitoring devices**		
3.1	☐Y ☐N	Is urgent CT scan available on arrival at the hospital?

3.1.1		If YES, what is the availability?
	☐Y	24 hours, 7 days of the week
	☐Y	Working hours only (8am-5pm)
	☐Y	Working hours (8am-5pm) and on – call after hours
3.2	☒Y ☐N	MR Imaging
3.3	☐Y ☐N	Is INR testing available at your site?
3.4	☐Y ☐N	Is emergency laboratory testing available at all times?
3.5	Which device do you use to monitor blood pressure?	
	☐Y	Automatic monitoring device
	☐Y	Electronic sphygmomanometer
	☐Y	Mercury sphygmomanometer
3.6	☐Y ☐N	Do you have adequate devices for blood pressure monitoring at your site?
3.7	☐Y ☐N	Do you have insulin bump or intravenous infusion bump at your site?
3.8	☐Y ☐N	Do you use fingertip glucometers to monitor blood glucose level?<br>***If not,*** please answer question 3.8.1
3.8.1	☐Y ☐N	Do you have any method to monitor blood glucose level?
		If yes, please specify: ________________________
**4. Management for ICH**		

4.1	*Are there local protocols (including management/monitoring) for any of the following situations? (tick all that apply)*	
	☐Y	Intensive blood pressure (BP) lowering
	☐Y	Reducing elevated blood glucose (BG) levels
	☐Y	Fever (Body temperature) control
	☐Y	Reversal of anticoagulation (if INR>1.5)
4.2	**Intensive blood pressure (BP) lowering**	
4.2.1	☐Y ☐N	Is there a policy indicating early commencement of intensive blood pressure lowering in admitted ICH patients at your hospital?
4.2.1.1		**If yes**, which is the first ward/unit to commence intensive BP lowering for ICH patients arrived hospital?
	☐Y	ED
	☐Y	Neurology Department
	☐Y	Neurosurgical Department
	☐Y	NICU
	☐Y	ICU
	☐Y	Acute Stroke Unit
	☐Y	Others, please specify________________________________
4.2.1.2	When will the intensive BP lowering commence after admission?	
	☐Y	<1 hour

	☐Y	1-6 hour
	☐Y	>6 hours
4.2.2	SBP: ______mmHg<br>DBP: ______mmHg	What is the recommended target of blood pressure lowering in your site? (SBP: systolic BP, DSP: diastolic BP)
4.2.3	________hrs	How long does it take to reach the target SBP?
4.2.4	What is the duration for maintaining the target SBP control?	
	☐Y	24 hour
	☐Y	48 hours
	☐Y	72 hours
	☐Y	7 days or before discharge
	☐Y	None of above, please specify______________________________
4.2.5	*What are the routine IV medications used in your department for BP lowering? (tick all that apply)*	
	☐Y	Urapidil
	☐Y	Metoprolol
	☐Y	Atenolol
	☐Y	Nicardipine
	☐Y	Clevidipine
	☐Y	Nimodipine
	☐Y	Nifedipine
	☐Y	Labetalol

	☐Y	Nitroprusside Sodium
	☐Y	Nitro-glycerine
	☐Y	Isosorbide Dinitrate
	☐Y	Frusemide
	☐Y	Mannitol
	☐Y	Furazosin
	☐Y	Hydralazine
	☐Y	Clonidine
	☐Y	Enalapril
	☐Y	Others, Please specify ____________________
4.2.6	*What are the routine oral medications used in your department for BP lowering? (tick all that apply)*	
	☐Y	Angiotensin II receptor antagonist
	☐Y	Diuretic
	☐Y	β blocker
	☐Y	Calcium channel blocker (CCB)
	☐Y	Angiotensin-converting-enzyme inhibitor (ACEI)
	☐Y	Central antihypertensive medications
	☐Y	Others, Please specify ____________________
4.3	**Blood glucose (BG) level control**	

4.3.1	Which kind of patients do you routinely manage for glycaemia in acute ICH phase? *(tick all that apply)*	
	☐Y	Diabetes patients only
	☐Y	Patients with increased blood glucose (BG) level, no matter diabetic or non-diabetic patients
	☐Y	None of above
4.3.2	D: ______mmol/L<br>N: ______mmol/L	When will you commence BG lowering for diabetes and Non-diabetic patients respectively? Specify BG level. (D: diabetes, N:non-diabetic patients)
4.3.3	D: ______mmol/L<br>N: ______mmol/L	When managing glycaemia level, what is the recommended target of BG level in your site for diabetes and non-diabetic patients respectively? Specify BG level.
4.3.4	What agents do you use for hyperglycaemic control in your site? *(tick all that apply)*	
	☐Y	Insulin
	☐Y	Oral hypoglycaemia agents
	☐Y	Never use
4.3.5	_____times per day	How often will BG level be monitored per day for patients with high level of BG?
4.4	**Fever (body temperature) control**	
4.4.1	______℃	For ICH patients, when will you commence fever control? Please specify level of body temperature.

4.4.2	☐Y ☐N	Do you routinely use paracetamol (oral or rectal administration) as antifebrile medication?
4.4.3	☐Y ☐N	Do you use IV infusion of 4℃ Normal Saline (0.9% NaCl) to control body temperature?
4.5	**Reversal of anticoagulation**	
4.5.1	INR ________	For ICH patients previously used anticoagulants, what level of INR will you commence reversal of anticoagulation?
4.5.2	|__|__|min	How long does it take to complete evaluation of coagulation function?
4.5.3	|__|__|min	How long does it take to cross-match and take blood?
4.5.4	Which agents do you usually used for anticoagulation reversal in your site?	
	☐Y	Vitamin K
	☐Y	Prothrombin Complex Concentrates (PCC)
	☐Y	Fresh Frozen Plasma (FFP)
**5. Epilepsy prevention after acute ICH**		
5.1	☐Y ☐N	Does your department conduct preventative treatment for patients with epilepsy after acute ICH?
5.2	If YES, what is the scope of prevention?	
	☐Y	Routine prevention for all ICH patients
	☐Y	Selective prevention
5.2.1		*If selective prevention*, what factors considered:

		☐Y	Haemorrhage in cerebral cortex
		☐Y	Age
		☐Y	Haematoma volume> 30ml
		☐Y	Surgery treatment
		☐Y	Others, please specify : ________
5.3	What medications will you use (please specify dosage, methods and period of treatment after the option, you can have multiple options)?		
	☐Y	Sodium Valproate	
	☐Y	Carbamazepine	
	☐Y	Levetiracetam	
	☐Y	Lamotrigine	
	☐Y	Diazepam	
	☐Y	Oxcarbazepine	
	☐Y	Topiramate	
**6. Ethics**			
6.1	☐Y ☐N	Does your hospital have any concerns of undertaking INTERACT3?<br>If YES, please specify : ____________________	
6.2	☐Y ☐N	Do you have any concerns of undertaking INTERACT3 in your department?<br>If YES, please specify: ____________________	

6.3	☐Y ☐N	Does ethics committee (EC) in your hospital accredited central ethic approval (enter into an agreement with an ethics committee from another institution to serve as its ethics committee of record)?
6.4	How long will take to get EC approval in your hospital?	
	☐Y	1 to 2 months
	☐Y	3 months
	☐Y	More than 3 months
